# Circulating growth factors data associated with insulin secretagogue use in women with incident breast cancer

**DOI:** 10.1016/j.dib.2017.02.038

**Published:** 2017-02-22

**Authors:** Zachary A.P. Wintrob, Jeffrey P. Hammel, George K. Nimako, Dan P. Gaile, Alan Forrest, Alice C. Ceacareanu

**Affiliations:** a State University of New York at Buffalo, Dept. of Pharmacy Practice, NYS Center of Excellence in Bioinformatics and Life Sciences, 701 Ellicott Street, Buffalo, NY 14203, United States; b Cleveland Clinic, Dept. of Biostatistics and Epidemiology, 9500 Euclid Ave., Cleveland, OH 44195, United States; c State University of New York at Buffalo, Dept. of Biostatistics, 718 Kimball Tower, Buffalo, NY 14214, United States; d The UNC Eshelman School of Pharmacy, Division of Pharmacotherapy and Experimental Therapeutics, Campus Box 7569, Chapel Hill, NC 27599, United States; e Roswell Park Cancer Institute, Dept. of Pharmacy Services, Elm & Carlton Streets, Buffalo, NY 14263, United States

**Keywords:** Growth factor, EGF, FGF, PDGF, HGF, TGF, VEGF, Insulin secretagogue, Breast cancer, Diabetes, Cancer outcomes, Cancer prognosis

## Abstract

Oral drugs stimulating insulin production may impact growth factor levels. The data presented shows the relationship between pre-existing insulin secretagogues use, growth factor profiles at the time of breast cancer diagnosis and subsequent cancer outcomes in women diagnosed with breast cancer and type 2 diabetes mellitus. A Pearson correlation analysis evaluating the relationship between growth factors stratified by diabetes pharmacotherapy and controls is also provided.

**Specifications Table**

**Table:** 

Subject area	Clinical and Translational Research
More specific subject area	Biomarker Research, Cancer Epidemiology
Type of data	Tables
How data was acquired	Tumor registry query was followed by vital status ascertainment, and medical records review
	Luminex®-based quantitation of growth factors (epidermal growth factor, fibroblast growth factor 2, vascular endothelial growth factor, hepatocyte growth factor, platelet-derived growth factor BB, and tumor growth factor-β) from plasma samples was conducted.
	A Luminex®200^TM^ instrument with Xponent 3.1 software was used to acquire all data
Data format	Analyzed
Experimental factors	Growth factors were determined from the corresponding plasma samples collected at the time of breast cancer diagnosis
Experimental features	The dataset included 97 adult females with diabetes mellitus and newly diagnosed breast cancer (cases) and 194 matched controls (breast cancer only). Clinical and treatment history were evaluated in relationship with cancer outcomes and growth factor profiles. A growth factor correlation analysis was also performed.
Data source location	United States, Buffalo, NY - 42° 53׳ 50.3592"N; 78° 52׳ 2.658"W
Data accessibility	The data is with this article

**Value of the data**

• This dataset shows the observed relationship between baseline insulin secretagogues use, circulating growth factor levels at the time of cancer diagnosis and breast cancer outcomes.
• Reported data may guide future studies evaluating pharmacotherapy-induced growth factor modulation in breast cancer.
• These observations can assist future study design in evaluating the relationship between diabetes pharmacotherapy safety and circulating growth factors levels at the time of cancer diagnosis.

## 1 Data

Reported data represents the observed association between use of insulin secretagogues preceding breast cancer and the growth factor profiles at the time of cancer diagnosis in women with diabetes mellitus (Table 1). Data in Table 2 includes the observed correlations between growth factors stratified by type 2 diabetes mellitus pharmacotherapy and controls. C-peptide correlation with each of the studied growth factors is presented in Table 2, however details regarding its determination from plasma, association with cancer outcomes and insulin secretagogues use has been already reported by us [2].

## 2 Experimental design, materials and methods

Evaluation of growth factor profile association with insulin secretagogue use and BC outcomes was carried out under two protocols approved by both Roswell Park Cancer Institute (EDR154409 and NHR009010) and the State University of New York at Buffalo (PHP0840409E). Demographic and clinical patient information was linked with cancer outcomes and growth factor profiles of corresponding plasma specimen harvested at BC diagnosis and banked in the Roswell Park Cancer Institute Data Bank and Bio-Repository.

### 2.1 Study population

As described in the original research article by Wintrob et al. [1], all incident breast cancer cases diagnosed at Roswell Park Cancer Institute (01/01/2003-12/31/2009) were considered for inclusion (*n*=2194). Medical and pharmacotherapy history were used to determine the baseline presence of diabetes.

### 2.2 Inclusion and exclusion criteria

Inclusion criteria were as follows: minimum 18 years of age at diagnosis, presence of pre-existing diabetes at breast cancer diagnosis, and having available banked treatment-naïve plasma specimens in the Institute׳s Data Bank and Bio-Repository. That is, the blood had to be collected prior to the initiation of any cancer-related therapy (surgery, radiation or pharmacotherapy).

Subjects were excluded if they were male, had prior cancer history or unclear date of diagnosis, incomplete clinical records, type 1 or unclear diabetes status. For a specific breakdown of excluded subjects, please see the original research article by Wintrob et al. [1].

A total of 97 female subjects with breast cancer and baseline diabetes mellitus were eligible for inclusion in this analysis.

### 2.3 Control-matching approach

Each of the 97 adult female subjects with breast cancer and diabetes mellitus (defined as “cases”) was matched with two other female subjects diagnosed with breast cancer, but without baseline diabetes mellitus (defined as “controls”). The following matching criteria were used: age at diagnosis, body mass index category, ethnicity, menopausal status and tumor stage (as per the American Joint Committee on Cancer). Some matching limitations applied [1].

### 2.4 Demographic and clinical data collection

Clinical and treatment history was documented by medical chart review. Vital status was obtained from the Institute׳s Tumor Registry, a local database updated biannually with data obtained from the National Comprehensive Cancer Networks’ Oncology Outcomes Database. Outcomes of interest were overall survival (death from breast cancer) and disease-free survival (breast cancer recurrence and/or death). Mean overall and disease-free survival were 113.3 and 107.3 months respectively, both with a minimum follow-up of 25.6 months. For additional details concerning data collection, specific definitions regarding censoring and drug use (including the number of insulin users per analyzed group), and a comprehensive demographic report, please see the original article by Wintrob et al. [1].

### 2.5 Plasma specimen storage and retrieval

All the plasma specimens retrieved from long-term storage were individually aliquoted in color coded vials labeled with unique, subject specific barcodes. Overall duration of freezing time was accounted for all matched controls ensuring that the case and matched control specimens had similar overall storage conditions. Only two instances of freeze-thaw were allowed between biobank retrieval and biomarker analyses: aliquoting procedure step and actual assay.

### 2.6 Luminex® assays

A total of 6 biomarkers (epidermal growth factor, fibroblast growth factor 2, vascular endothelial growth factor, hepatocyte growth factor, platelet-derived growth factor BB, and tumor growth factor-β) were quantified according to the manufacturer protocol. The following Luminex® biomarker panels were utilized in this study: TGFB-64K (tumor growth factor-β), HCYTOMAG-60K (platelet-derived growth factor BB), and HAGP1MAG-12K (epidermal growth factor, fibroblast growth factor 2, vascular endothelial growth factor, and hepatocyte growth factor) produced by Millipore Corporation, Billerica, MA. C-peptide determinations were done according to the manufacturer protocol as previously reported [2].

### 2.7 Biomarker-pharmacotherapy association analysis

Biomarker cut-point optimization was performed for each analyzed biomarker. Biomarker levels constituted the continuous independent variable that was subdivided into two groups. Cut-point selection was determined by p-value optimization using the log rank method with respect to survival (both overall and disease-free) as the dependent variable with the condition of a minimum biomarker group size of 10 patients. The results of this analysis yielded the cut-point for each biomarker that would provide the most significant separation of a Kaplan-Meier survival probability curve by assigning the subject to their respective biomarker category, specifically above or below the identified cut-point. Thus identifying potential biomarker ranges associated with poorer outcomes, specifically, ranges associated with a lower survival probability. Quartiles were also constructed. The resultant biomarker categories were then ztested for association with type 2 diabetes mellitus therapy and controls by Fisher׳s exact test. The continuous biomarker levels were also tested for association with diabetes therapy and controls across groups by the Kruskal–Wallis test and pairwise by the Wilcoxon rank sum. Multivariate adjustments were performed accounting for age, tumor stage, body mass index, estrogen receptor status, and cumulative comorbidity. The biomarker analysis was performed using R Version 2.15.3. Please see the original article for an illustration of the analysis workflow [1].

Correlations between biomarkers stratified by type 2 diabetes mellitus pharmacotherapy and controls were assessed by the Pearson method. Correlation models were constructed both with and without adjustment for age, body mass index, and the combined comorbidity index. Correlation analyses were performed using SAS Version 9.4.

## Funding Sources

This research was funded by the following grant awards: Wadsworth Foundation Peter Rowley Breast Cancer Grant awarded to A.C.C. (UB Grant number 55705, Contract CO26588).

## Figures and Tables

**Table 1:** Growth factor associations with cancer outcomes and insulin secretagogues use.

Biomarker	Biomarker grouping	Concentration	Control	No Secretagogue	Any Secretagogue	Unadjusted *P*-value (MVP)			
						p^1^	p^2^	p^3^	Global test
EGF (ng/ml)	Median, ng/ml (25th–75th)	–	20.26 (12.25–37.04)	29.60 (18.76–56.42)	26.63 (15.35–53.77)	0.002 (0.002)	0.041 (0.400)	0.330 (0.120)	0.003 (0.007)
								
	Quartiles	1.60–13.61	57 (29.4%)	6 (12.8%)	10 (20.0%)	0.020	0.280	0.740	0.070
		13.79–23.29	51 (26.3%)	10 (21.3%)	12 (24.0%)				
		23.70–44.72	47 (24.2%)	13 (27.7%)	12 (24.0%)				
		45.35–382.99	39 (20.1%)	18 (38.3%)	16 (32.0%)				
								
	OS-Based Optimization	1.60–113.10	189 (97.4%)	42 (89.4%)	47 (94.0%)	0.027 (0.080)	0.210 (0.830)	0.480 (0.440)	0.035 (0.160)
		**116.01–382.99**^a^	5 (2.6%)	5 (10.6%)	3 (6.0%)				
								
	DFS-Based Optimization	**1.60–5.20**^a^	12 (6.2%)	1 (2.1%)	4 (8.0%)	0.470 (0.220)	0.750 (0.380)	0.360 (0.110)	0.490 (0.240)
		5.39–382.99	182 (93.8%)	46 (97.9%)	46 (92.0%)				
									
FGF-2 (pg/ml)	Median, pg/ml (25th–75th)	–	16.15 (4.32–34.43)	30.58 (7.13–49.11)	14.66 (3.20–42.68)	0.048 (0.034)	0.730 (0.600)	0.230 (0.280)	0.150 (0.080)
								
	Quartiles	1.60–4.18	49 (25.3%)	10 (21.3%)	14 (28.0%)	0.220	0.560	0.620	0.430
		4.76–17.34	51 (26.3%)	9 (19.1%)	13 (26.0%)				
		17.51–39.78	52 (26.8%)	11 (23.4%)	9 (18.0%)				
		40.30–1147.64	42 (21.6%)	17 (36.2%)	14 (28.0%)				
								
	OS-Based Optimization	**1.60–10.15**^a^	72 (37.1%)	15 (31.9%)	19 (38.0%)	0.510 (0.540)	0.910 (0.830)	0.530 (0.870)	0.780 (0.780)
		10.21–1147.64	122 (62.9%)	32 (68.1%)	31 (62.0%)				
								
	DFS-Based Optimization	**1.60–14.61**^a^	87 (44.8%)	17 (36.2%)	25 (50.0%)	0.280 (0.330)	0.510 (0.400)	0.170 (0.160)	0.380 (0.290)
		14.68–1147.64	107 (55.2%)	30 (63.8%)	25 (50.0%)				
									
HGF (pg/ml)	Median, pg/ml (25th–75th)	–	289 (129–439)	347 (193–507)	348 (136–576)	0.160 (0.590)	0.220 (0.980)	0.910 (0.280)	0.240 (0.660)
								
	Quartiles	13.02–130.22	50 (25.8%)	11 (23.4%)	12 (24.0%)	0.670	0.021	0.350	0.110
		130.72–312.56	52 (26.8%)	10 (21.3%)	11 (22.0%)				
		314.96–472.00	53 (27.3%)	13 (27.7%)	7 (14.0%)				
		505.37–6728.77	39 (20.1%)	13 (27.7%)	20 (40.0%)				
								
	OS-Based Optimization	13.02–1148.76	188 (96.9%)	45 (95.7%)	48 (96.0%)	0.660 (0.770)	0.670 (0.960)	1.000 (0.840)	0.700 (0.960)
		**1169.11–6728.77**^a^	6 (3.1%)	2 (4.3%)	2 (4.0%)				
								
	DFS-Based Optimization	13.02–919.06	185 (95.4%)	44 (93.6%)	44 (88.0%)	0.710 (0.770)	0.090 (0.250)	0.490 (0.460)	0.170 (0.640)
		**920.11–6728.77**^a^	9 (4.6%)	3 (6.4%)	6 (12.0%)				
									
PDGF-BB (pg/ml)	Median, pg/ml (25th–75th)	–	2055 (615–5402)	1341 (309–2802)	1105 (205–3211)	0.100 (0.043)	0.037 (0.015)	0.710 (0.850)	0.053 (0.022)
								
	Quartiles	60–414	43 (22.2%)	13 (27.7%)	17 (34.0%)	0.610	0.210	0.800	0.460
		440–1618	47 (24.2%)	12 (25.5%)	14 (28.0%)				
		1660–4332	49 (25.3%)	13 (27.7%)	10 (20.0%)				
		4355– 15,480	55 (28.4%)	9 (19.1%)	9 (18.0%)				
								
	OS-Based Optimization	**60–2687**^a^	109 (56.2%)	34 (72.3%)	35 (70.0%)	0.046 (0.014)	0.080 (0.035)	0.800 (0.940)	0.046 (0.017)
		2694– 15,480	85 (43.8%)	13 (27.7%)	15 (30.0%)				
								
	DFS-Based Optimization	**60– 10,400**^a^	186 (95.9%)	44 (93.6%)	49 (98.0%)	0.450 (0.690)	0.690 (0.710)	0.350 (0.450)	0.490 (0.690)
		10,944– 15,480	8 (4.1%)	3 (6.4%)	1 (2.0%)				
									
TGF-β (pg/ml)	Median, pg/ml (25th–75th)	–	3007 (1996–4053)	4063 (2678–4872)	3425 (2417–4414)	0.013 (0.250)	0.070 (0.600)	0.450 (0.660)	0.017 (0.480)
								
	Quartiles	453–2151	57 (29.4%)	7 (14.9%)	9 (18.0%)	0.060	0.110	0.440	0.052
		2155–3157	52 (26.8%)	11 (23.4%)	10 (20.0%)				
		3183–4303	43 (22.2%)	11 (23.4%)	18 (36.0%)				
		4311– 12,026	42 (21.6%)	18 (38.3%)	13 (26.0%)				
								
	OS-Based Optimization	**453–5545**^a^	176 (90.7%)	39 (83.0%)	43 (86.0%)	0.130 (0.220)	0.330 (0.890)	0.680 (0.320)	0.260 (0.480)
		5557– 12,026	18 (9.3%)	8 (17.0%)	7 (14.0%)				
								
	DFS-Based Optimization	**453–1881**^a^	42 (21.6%)	6 (12.8%)	6 (12.0%)	0.180 (0.210)	0.130 (0.470)	0.910 (0.800)	0.160 (0.370)
		1907– 12,026	152 (78.4%)	41 (87.2%)	44 (88.0%)				
									
VEGF (pg/ml)	Median, pg/ml (25th–75th)	–	95.07 (40.78–189.51)	124.31 (59.38–308.06)	87.25 (42.25–192.36)	0.110 (0.190)	0.780 (0.870)	0.260 (0.380)	0.270 (0.400)
								
	Quartiles	1.60–43.56	52 (26.8%)	8 (17.0%)	13 (26.0%)	0.210	0.680	0.120	0.320
		44.52–97.48	51 (26.3%)	9 (19.1%)	16 (32.0%)				
		97.87–192.64	45 (23.2%)	16 (34.0%)	8 (16.0%)				
		194.47–4197.81	46 (23.7%)	14 (29.8%)	13 (26.0%)				
								
	OS-Based Optimization	**1.60–37.94**^a^	45 (23.2%)	7 (14.9%)	10 (20.0%)	0.220 (0.150)	0.630 (0.810)	0.510 (0.570)	0.450 (0.370)
		38.42–4197.81	149 (76.8%)	40 (85.1%)	40 (80.0%)				
								
	DFS-Based Optimization	**1.60–37.94**^a^	45 (23.2%)	7 (14.9%)	10 (20.0%)	0.220 (0.150)	0.630 (0.810)	0.510 (0.570)	0.450 (0.370)
		38.42–4197.81	149 (76.8%)	40 (85.1%)	40 (80.0%)				

Unadjusted *p*-values: p^1^, compares *no secretagogue versus control*; p^2^, compares *any secretagogue versus control*; p^3^, compares *any secretagogue versus no secretagogue* (as per Kruskal–Wallis test); global test, compares *all categories* (as per Wilcoxon, type 3 error test); MVP, denotes the *p*-value of each multivariate adjusted analysis corresponding to the earlier described unadjusted analyses. For more information, please see Section 2.7 below and our previously published analysis work flow^1^. MVP= *p*-value of the multivariate adjusted analysis. Epidermal growth factor (EGF), fibroblast Growth Factor 2 (FGF-2), hepatocyte growth factor (HGF), platelet-derived growth factor BB (PDGF-BB), tumor growth factor (TGF), vascular endothelial growth factor (VEGF).

a Overall survival (OS)- and disease-free survival (DFS)-optimized growth factor ranges associated with poorer outcomes (i.e. the group with a lower survival probability) are represented in bold.

**Table 2:** Growth factor correlations by secretagogues use.





Significant correlations are displayed in bolded text. The differences that are only significant in either adjusted or unadjusted correlations are further denoted by an outline. Epidermal growth factor (EGF), fibroblast Growth Factor 2 (FGF-2), hepatocyte growth factor (HGF), platelet-derived growth factor BB (PDGF-BB), tumor growth factor (TGF), vascular endothelial growth factor (VEGF).
